# Raising the bar on sex and gender reporting in research

**DOI:** 10.1038/s41467-022-30398-1

**Published:** 2022-05-18

## Abstract

For nearly a decade, Nature Portfolio journals have asked for information about sex and gender in research studies, and more recently, we have also encouraged authors to use the Sex and Gender Equity in Research (SAGER) guidelines in their work. We are now updating our guidance and practice so that authors are more clearly and actively encouraged to report on select items within the SAGER guidelines.

Funders and journals have had policy initiatives in place for many years and the number of research studies that include sex and gender as key variables has increased significantly across most biological disciplines in the past decade. However important gaps remain, including presentation of data that is disaggregated by sex and gender, a reliance on a single sex or gender without appropriate justification, and a lack of appropriate sex and gender analysis (Refs. ^1–3^) Analysis of the journals’ efforts, introduced in 2013 through *Nature’s* reporting checklist, also found sex was reported in 52% of the studies in Nature journals versus 36% in non-Nature journal studies (Ref. ^4^).

The emphasis of these new policy expectations is on promoting transparency in study design and improved reporting, and over time, we hope to see integration of sex and gender analysis in study design by default

Given the importance of understanding sex and gender as variables that affect rigour, generalizability and translation of research findings, we are making changes that we hope will support authors and reviewers in providing and vetting this information during peer review. These changes apply to studies with human research participants, vertebrate animals and cell lines, where sex and gender is an appropriate consideration, and will come into effect from June 2022. First, our updated guidance more clearly focuses on the key items from the SAGER guidelines, which we are introducing in a new section on “Reporting on Sex and Gender” in the Nature Life Science Reporting Summary. This new section asks authors to state whether and how sex and gender were considered in study design, indicate if no sex and gender analysis were carried out and clarify why, note in the title and/or abstract if findings apply to only one sex or gender, and finally, provide data disaggregated by sex and gender where this information has been collected and informed consent for reporting and sharing individual-level data has been obtained. We hope that requiring these details in a structured manner through established implementation routes will induce more authors to provide this information in published articles. Second, Nature Communications, together with Nature Cancer, Nature Medicine and Nature Metabolism will be running a pilot raising awareness of our updated recommendations in letters to authors and reviewers during peer review and actively asking authors to provide disaggregated data when available. The pilot will allow us to better understand the degree to which these considerations are already part of study design, data collection and analysis for the research we publish; and to evaluate author and reviewer reception to our updated guidance and challenges in its implementation, so that we may iterate on these recommendations as we learn through experience.

The emphasis of these new policy expectations is on promoting transparency in study design and improved reporting, and over time, we hope to see integration of sex and gender analysis in study design by default. Alongside the push for inclusion of sex and gender in study design, analysis and reporting, we also urge care and due caution in communicating findings about sex and gender, so as to avoid inadvertent and harmful effects of research findings especially where there is the potential for societal and public policy impact. We look forward to engaging more deeply with our authors, referees and readers as we roll out these updated policies.

## References

[1] Woitowich, N. C. et al. A 10-year follow-up study of sex inclusion in the biological sciences. *eLife* **9**, e56344 (2020). 10.7554/eLife.56344 PMC7282816 32513386

[2] Brady, E. et al. Lack of consideration of sex and gender in COVID-19 clinical studies. *Nat. Commun*. **12**, 4015 (2021). 10.1038/s41467-021-24265-8 PMC8260641 34230477

[3] Rechlin, K. K. et al. An analysis of neuroscience and psychiatry papers published from 2009 and 2019 outlines opportunities for increasing discovery of sex differences. *Nat. Commun.* **13**, 2137 (2022). 10.1038/s41467-022-29903-3 PMC9018784 35440664

[4] NPQIP Collaborative group. Did a change in Nature journals’ editorial policy for life sciences research improve reporting? *BMJ Open Science* **3**, e000035 (2019). 10.1136/bmjos-2017-000035 PMC8647608 35047682

